# FEMSEC—thematic issue “Rhizosphere—a One Health concept”

**DOI:** 10.1093/femsec/fiae136

**Published:** 2024-10-29

**Authors:** Anton Hartmann, Luz de Bashan, Birgit Wassermann, Marcus A Horn, Angela Sessitsch

**Affiliations:** Faculty of Biology, Microbe-Plant Interactions, Ludwig-Maximilian-University Munich, Grosshaderner Str. 2, D-82152 Planegg/Martinsried, Germany; Bashan Institute of Science, Auburn, AL 36853, United States; Institute of Environmental Biotechnology, Graz University of Technology, Petersgasse 12/1, A-8010 Graz, Austria; Institute for Microbiology, Leibniz-University of Hannover, Herrenhaeuser Str. 2, D-30419 Hannover, Germany; Bioresources Unit, AIT Austrian Institute of Technology, A-1210 Vienna, Austria

## Abstract

The strength of the microbial biogeographic patterns decreased along the increasing gradient of habitat specificity (from sediment to gut tissue) provided by a benthic sea urchin in the Southern Ocean.

Professor Lorenz Hiltner, the pioneer of the “rhizosphere concept,” died about 100 years ago in June 1923 only 62 years old in Munich, Germany. In 1904, he had stated that the nutrition of plants is in general dependent upon the composition of microbes in the rhizosphere—the zone around the root attracting useful bacteria by their exudates; however also plant pathogens responding to the root exudates are attracted (Hiltner 1904, Hartmann et al. 2008). This proposal of root/microbe/soil interactions as central communication sphere determining the health of plants provided the blueprint for major scientific developments about the intimate role of microbes for plant nutrition and health. Although he could not reveal many details of microbe–plant interactions using the scientific tools and approaches available at his time, the progress since then enabled to deepen the understanding of microbe–plant interactions in the rhizosphere to multiple interactions of microbiomes (including viruses, fungi, and bacteria). Thus, the rhizosphere interaction of microbes with plants toward their health can be regarded as initial model of microbial influences with all organisms in the “One Health concept” (Banerjee and van der Heijden 2023).

The thematic issue “Rhizosphere—a One Health concept” focuses specifically on the interaction of microbes with plant roots, presenting a collection of perspective reviews and original publications documenting the present state of the art. The holobiont theory summarizes the manifold symbiotic-like associations of biotic partners. Many ecological and genetic features of microbiome–plant interactions reveal principles of a shared governance between hosts and microbes (Berg et al. 2024). The authors point out knowledge gaps that arise when integrating plant holobionts in a broader perspective of holobiome interactions in a one environmental or planetary health concept. In many aspects, a deeper understanding is needed for the prediction and control of microbiomes to improve plant, human, and environmental health. One central aspect is an improved knowledge of basic molecular mechanisms in cross-kingdom interactions. Thus, the metabolomic potential of the partners within holobionts has key importance. Since many microbial partners organize their metabolic activities using quorum-sensing (QS) signaling of beneficial as well as pathogenic bacteria, these metabolites play important roles for holobionts (Hartmann et al. 2024). Focusing on QS-autoinducers *N*-acylhomoserine lactones and alternative autoinducers (AI-2) of Gram-negative bacteria as well as autoinducer peptides (AI-P) of Gram-positive bacteria, different interactions with the hosts (plant and human) are reviewed. In most studies on holobiont interactions in the framework of One Health, the role of viruses has been mostly neglected. This gap is narrowed by the review of Wang et al. (2024), which summarizes the important diversity and ecological roles of phages in soil and rhizosphere microbiomes in terms of nutrient cycling, top-down diversity regulation, and pathogen suppression. Bacterial evolution is driven by promoting horizontal gene transfer through phage transduction encoding auxiliary metabolic and resistance genes increasing bacterial fitness. This particular aspect is documented by the model study of Chen et al. (2023) on the effect of application of chicken manure containing antibiotic resistance genes on the microbial community structure in the rhizosphere of *Cinnamorium camphora* in the presence of several antibiotics.

A number of publications focus on the role of plants on the rhizosphere microbiome. The study of Byers et al. (2023) identifies the role of plant species identity and plant-induced changes in soil physico-chemistry on the assembly of root-associated soil microbiome. By their analysis of root-associated rhizosphere soil they did not find an influence of plant functional traits and phylogeny, while a symbiotic footprint was found by Hartman et al. (2023), when analysing plant root and rhizoplane microbiomes of symbionts. Thus, the abiotic condition of the soil environment is of greater influence on microbial communities assembled in the root-associated soil environment and it depends on the particular rhizosphere sampling procedure, which type of root-associated influence on microbiomes can be found (Behr et al. 2024).

In a truly interdisciplinary field study by Behr et al. (2024) the influence of agricultural practices on rhizosphere microbiome, plant–microorganism interactions, and crop performance of winter wheat was studied using root observation windows comparing long-term mouldboard plough versus cultivator tillage practices. *In situ* root growth characteristics and rhizosphere metabolite composition were monitored together with soil and rhizosphere microbiome structure, while above ground shoot biomass, plant nutritional status, and physiological stress indicators assessed plant health. The type of tillage practice was found as a major driver of crop performance (plant growth and stress resilience) and root activities (deposits and rhizosphere microbiome–plant interactions).

Drought is a major stressor to soil communities and the intensification of climate change is predicted to increase water stress worldwide. In the study of de Souza et al. (2024), the interaction between plant species richness and rhizosphere soil microbial communities was investigated in successive simulated summer drought periods over a period of 9 years. A significant effect of plant species richness on the microbial community composition was observed by drought conditions at each plant richness level and accordingly affected microbial taxa could be identified.

Increased salinity affects the rhizosphere microbiome, which was shown by Dubey et al. (2023). Using a multipassaging approach, the authors demonstrated that the rhizosphere fungal community undergoes dynamic changes facilitating salt stress mitigation in *Vigna radiata* plants. The application of acclimatized rhizosphere community additionally enhanced plant biometrics and reduced salt stress marker levels in the plants. Another aspect of influence of the rhizosphere microbiome assembly for plant health was studied in a 150 years chronosequence of the colonization of three pioneer plants in the Hallstaedter glacier forefield (Wikacsono et al. 2024). Glaciation was demonstrated as important factor shaping the rhizosphere microbiome toward nutrient uptake and stress protection by the colonization of ubiquitous and cosmopolitan bacteria from the surrounding soil. Along the chronosequence, a decreasing microbial richness but increasing specificity characterized the plant-associated bacterial community during long-term establishment of the plant hosts. A most interesting aspect for role of the rhizosphere microbiome in improving plant productivity was studied by Martins et al. (2024), comparing 51 potato cultivars under similar greenhouse conditions using a metabarcoding approach. Significant differences were detected when grouping the cultivars according to their plant growth characteristics. The genera *Arthrobacter* and *Massilia*, known for higher indole acetic acid and siderophore production, were more abundant. Bacterial co-occurrence networks were larger in the rhizosphere of cultivars with improved growth characteristics and characterized by a distinctive combination of plant beneficial proteobacteria and actinobacteria along with a module of diazotrophic bacteria. In contrast, the networks in low performing cultivars revealed the lowest nodes, robustness, and highest average path length resulting in reduced microbial associations, which could limit effective plant growth-promoting effects. This offers insights in future management practices (Martins et al. 2024).

Different insight in the complex interactions between plants and their associated microorganisms for optimizing plant health is provided by the study of Vlasselaer et al. (2024) on the microbiome of hydroponically cultivated lettuce. They additionally investigated the impact of the oomycete pathogen *Phytophthora cryptogea* on plant-associated microbiomes. Amplicon sequencing of the 16S rRNA gene revealed significant alterations in the rhizosphere bacterial community upon *P. cryptogea* infection. In symptomatic and asymptomatic plants, significant differences were revealed by permutational multivariate analysis of variance. In particular, members of *Pseudomonas* and *Flavobacterium* were enriched in the rhizosphere of symptomatic plants.

In the Moroccan endemic plant *Vachellia gummifera* growing under extreme desert conditions, the plant growth-promoting activities of 500 bacteria were screened for phosphate solubilization potential, siderophore, and auxin production by Bennis et al. (2023). Members of the genera *Enterobacter* and *Pseudomonas* were shown to cause 200% higher plant shoot weight and 139% enhanced root length upon inoculation. Some combinations also improved protein and chlorophyll content in inoculated plants thereby improving the plant’s forage value.

The enrichment of rhizosphere bacteria improving plant performance under salinity and drought conditions was achieved through an *ex situ* plant trapping method to collect the culturable microbial diversity in soils from harsh and remote areas (Amenta et al. 2024). They used *Oryza sativa* and *Triticum durum* as recruiters, while soil surrounding roots of *Oryza glaberrima* plants from remote regions of Mali (West Africa) was used as growth substrate. The endophytic community of both recruiter plants contained as dominant genera *Bacillus, Kosakonia*, and *Enterobacter*. These isolates showed halotolerance, inorganic phosphate-solubilizing, and N_2_-fixing activities. The characterized bacteria with beneficial traits could be used for the improvement of rice and wheat under adverse environmental conditions (Amenta et al. 2024).

In salt-affected soil in Germany near Giessen, a plant growth-promoting diazotrophic bacterium *Hartmannibacter diazotrophicus* E19^T^ had been isolated (Suarez et al. 2014). Having the complete genome sequenced, details of functions potentially involved in rhizosphere/root colonization, such as those involved in quorum sensing, chemotaxis, secretory protein synthesis, antibiotic resistance, iron import, and general stress response were identified. Strain-specific primers were developed to monitor *H. diazotrophicus* E19 on the roots (Quiroga et al. 2024). Using these primers, early and long-term persistence and quantification of E19 under field conditions were revealed. In this way, a correlation between the number of copies of E19 and observed stimulation effects on yield parameters of winter wheat and summer barley were found. Especially, since the bacterium is salt tolerant, the colonization of roots was improved at elevated salt concentrations (Quiroga et al. 2024). The effect of inoculation of *H. diazotrophicus* E19 on the rhizosphere microbiome and its possible integration into interacting networks still needs to be studied.

In conclusion, the publications collected under the Special Thematic “Rhizosphere—a One Health concept” reveal different aspects of microbe–plant interactions. However, the “Rhizosphere—One Health concept” is surely going beyond relevance for plant health and has important implications for environmental and animal/human health. The One Health role of microbes with beneficial but also pathogenic potential for holobiont interaction networks presents a blueprint for complex microbe–host/environment interactions for even One Health global scale.
