# Correction to: FEMSEC—thematic issue “Rhizosphere—a One Health concept”

**DOI:** 10.1093/femsec/fiae165

**Published:** 2025-01-28

This is a correction to: Anton Hartmann, Luz de Bashan, Birgit Wassermann, Marcus A Horn, Angela Sessitsch, FEMSEC—thematic issue “Rhizosphere—a One Health concept”, *FEMS Microbiology Ecology*, Volume 100, Issue 11, November 2024, fiae136, https://doi.org/10.1093/femsec/fiae136

In the originally published version, the year of Professor Lorenz Hiltner's death was given incorrectly. This has been corrected.

